# Factors associated with postpartum depression symptoms following antepartum hospitalization

**DOI:** 10.1002/pmf2.70319

**Published:** 2026-05-22

**Authors:** Alison N. Goulding, Daniel Palacios, Sukru Aras, Hu Chen, Sasidhar Pasupuleti, Marika Toscano, Nicole Cirino, Israel C. Christie, Zhandong Liu, Emily S. Miller, Terri L. Fletcher

**Affiliations:** ^1^ Division of Maternal‐Fetal Medicine Department of Obstetrics and Gynecology Baylor College of Medicine Houston Texas USA; ^2^ VA HSR&D Houston Center of Innovations in Quality Effectiveness and Safety Michael E. DeBakey VA Medical Center Houston Texas USA; ^3^ Graduate School of Biomedical Sciences Program in Quantitative and Computational Biosciences Baylor College of Medicine Houston Texas USA; ^4^ Department of Pediatrics Baylor College of Medicine Houston Texas USA; ^5^ Jan and Dan Duncan Neurologic Research Institute Texas Children's Hospital Houston Texas USA; ^6^ Data Science Center Texas Children's Hospital Houston Texas USA; ^7^ Division of Maternal‐Fetal Medicine Department of Gynecology and Obstetrics Johns Hopkins University Baltimore Maryland USA; ^8^ Division of Reproductive Psychiatry Department of Obstetrics and Gynecology Baylor College of Medicine Houston Texas USA; ^9^ Menninger Department of Psychiatry and Behavioral Sciences Baylor College of Medicine Houston Texas USA; ^10^ Division of Health Services Research Department of Medicine Baylor College of Medicine Houston Texas USA; ^11^ Division of Maternal‐Fetal Medicine Department of Obstetrics and Gynecology Warren Alpert Medical School of Brown University Providence Rhode Island USA; ^12^ Women & Infants Hospital of Rhode Island Providence Rhode Island USA; ^13^ VA South Central Mental Illness Research Education and Clinical Center Houston Texas USA

**Keywords:** antenatal, antepartum, depression, mental health, perinatal, postpartum, pregnancy

## Abstract

**Background:**

Hospitalized antepartum patients are at increased risk for postpartum depression (PPD). Developing approaches to identify those at highest risk for PPD would enable timely and targeted intervention.

**Objective:**

We aimed to identify factors associated with the development of PPD symptoms in hospitalized antepartum patients and to assess their predictive utility.

**Study design:**

This retrospective cohort study included pregnant individuals hospitalized in a regional referral center due to medical or obstetric complications between 2012 and 2025. Data were extracted from the electronic health record, including demographics, medical and obstetric history, hospitalization characteristics, and postpartum Edinburgh Postnatal Depression Scale (EPDS) scores collected within 8 weeks of delivery. Primary outcome was EPDS score ≥10, indicating PPD symptoms. We performed bivariate analyses and multivariable logistic regression modeling. Receiver operating characteristic (ROC) curve analyses assessed model discriminatory ability.

**Results:**

Among 4161 included hospitalized antepartum patients, 1291 (31%) reported PPD symptoms within 8 weeks of delivery. Multivariable logistic regression modeling showed that certain demographic and clinical factors were associated with PPD symptoms: single or other marital status, pregestational diabetes, multiple chronic medical conditions, prior anxiety or depression, admission gestational age <28 weeks, preterm prelabor rupture of membranes, placenta accreta spectrum, and pharmacologic treatment for anxiety or depression during antepartum hospitalization. The strongest predictors for the presence of PPD symptoms were prior history of anxiety or depression (adjusted odds ratio [aOR], 2.04; 95% confidence interval [CI], 1.75–2.38) and pharmacologic treatment for anxiety or depression during antepartum hospitalization (aOR, 2.33; 95% CI, 1.86–2.90). Discriminatory ability of the multivariable model was fair, with area under the receiver operating characteristic curve (AUC) of 0.67 (95% CI, 0.65–0.69), positive predictive value of 58%, and negative predictive value of 72%.

**Conclusions:**

One in three individuals who experienced antepartum hospitalization at our referral center went on to report PPD symptoms. While certain demographic and clinical factors were associated with PPD symptoms, their overall ability to accurately identify those at increased risk was limited, reducing their utility in guiding targeted interventions. These findings support that postpartum mental health services are broadly needed for individuals who experience antepartum hospitalization, highlighting the need for universal mental health service provision for this high‐risk population.

## 1 INTRODUCTION

Mental health conditions such as depression and anxiety disorders are common during the perinatal period, affecting more than one in five individuals [1, 2]. Once diagnosed, many perinatal patients unfortunately do not receive any form of mental health treatment, either psychotherapy or psychopharmacotherapy, despite the existence of these evidence‐based treatments [3, 4]. It has been repeatedly demonstrated that untreated mental health conditions are associated with adverse maternal outcomes, including worsened chronic conditions, increased substance use, and increased maternal morbidity and mortality [5, 6]. Mental health conditions are a leading cause of maternal mortality in the United States, contributing to over 20% of pregnancy‐related deaths [7, 8]. Untreated perinatal mental health conditions can also contribute to adverse obstetric (fetal growth restriction, preterm birth, and stillbirth) [6, 9, 10] and child (impaired neurodevelopment and behavior) [11, 12] outcomes. The societal costs of untreated perinatal mental health conditions are substantial, with estimates of total costs reaching $14.2 billion annually in the United States [13].

Hospitalized antepartum patients, defined as individuals hospitalized prior to delivery for obstetric or medical complications, are a special population at increased risk for perinatal mental health conditions. One in three hospitalized antepartum patients screen positive for depression or anxiety during their hospitalization, approximately twice the prevalence in the general obstetric population [14]. This high‐risk population faces numerous stressors that can harm mental health. The experience of pregnancy complications itself imparts increased risk for depression and anxiety symptoms, independent of hospitalization [15]. These risks are further exacerbated by the stress, social isolation, and sleep disruption that can occur during hospitalization [16]. Most hospitalized antepartum patients will ultimately give birth preterm, and parenting a preterm baby with associated medical complications can contribute to adverse mental health outcomes [17, 18]. In our prior qualitative work [19], hospitalized antepartum patients described emotional distress associated with their hospitalization and identified a range of stressors depending on individual life circumstances, including finances, additional childcare needs, employment disruption, and new worries about maternal and fetal health. While representing a small proportion of pregnant patients, with estimates ranging from 1% to 3% of the general obstetric population [20, 21], hospitalized antepartum patients represent a unique, high‐risk population who warrant additional attention and healthcare resources.

The postpartum period is a time of heightened vulnerability for mental health complications, as new mothers navigate substantial biological, psychological, and social changes, often with limited support [22, 23]. The majority of pregnancy‐related deaths in the United States, including those caused by mental health conditions, occur during the postpartum period and are preventable [7, 8]. During the critical postpartum time, patients face unique and formidable system‐level barriers to accessing mental health care [24], driven in part by the severe shortage of mental health professionals [25, 26, 27].

Specific to hospitalized antepartum patients, there are few studies examining the trajectory of mental health symptoms and postpartum outcomes [14]. Available data suggest that hospitalized antepartum patients are at increased risk for postpartum depression (PPD) [28, 29] and its associated harms for mothers (decreased quality of life, impaired physical and psychological health, and relationship difficulties) and children (impaired cognitive, language, and motor development, breastfeeding difficulties, and disrupted maternal‐infant bonding) [30]. However, existing studies are limited by small cohort sizes and resultant generalizability concerns. Additionally, despite recommendations to universally screen for perinatal mental health conditions [31], screening rates are low in all settings [24, 32], making it challenging to appropriately direct mental health resources. Given these constraints, our limited mental health resources should be directed to those at the highest risk of mental health complications, including PPD. Developing approaches to identify those at highest risk of PPD would enable timely and targeted intervention among postpartum individuals who experienced antepartum hospitalization, and such approaches could later be expanded to broader perinatal populations. In this study, we aimed to identify factors associated with the development of PPD symptoms in hospitalized antepartum patients and to assess their predictive utility.

## 2 MATERIALS AND METHODS

This retrospective cohort study included pregnant individuals hospitalized for management of medical or obstetric complications at a regional referral center in Houston, TX, from March 2012 to March 2025. Individuals hospitalized on the antepartum unit for at least 48 h were included. For participants with multiple antepartum hospitalizations, only the initial hospitalization was included in this study. Similarly, if a patient had multiple pregnancies during the study period, only data from their first pregnancy was considered. Data extracted from the electronic health record included demographics, medical and obstetric history (determined by diagnosis codes), hospitalization characteristics (including gestational age at admission and medications prescribed throughout admission), and postpartum Edinburgh Postnatal Depression Scale (EPDS) scores collected within 8 weeks of delivery at outpatient postpartum visits. Patients without postpartum EPDS scores were excluded. If there were multiple EPDS scores for a single participant within 8 weeks of delivery, the highest EPDS score was included in analyses, in order to capture peak depressive symptoms reported in the postpartum period. Even if not sustained, elevated depressive symptoms at any point during the postpartum period can have significant adverse impacts on women and their families. The EPDS is a validated screening instrument [33] that is commonly used in perinatal populations. It screens for both depression and anxiety symptoms and includes a question about self‐harm. The EPDS has excellent overall test characteristics for the identification of depression, with results comparable to a structured clinical interview for DSM‐5 major depression diagnosis [34]. The operating characteristics of the EPDS are similar to those of the other widely used screening instrument for perinatal depression, the Patient Health Questionnaire‐9 (PHQ‐9) [35]. The primary outcome for this study was EPDS score ≥10, indicating the presence of PPD symptoms within the last 2 weeks. While both EPDS threshold values of 10 and 13 have been used in prior studies [36], we elected to use a threshold of 10 or higher to capture mild depressive symptoms [31], which can have significant impacts on women and their families. Additionally, selection of this lower threshold score maximizes sensitivity of this screening tool to detect major depression [37].

We initially performed descriptive statistics and data visualization. Missing data for numerical variables in our cohort was addressed using median imputation. Normality testing was performed using both visual assessment and formal testing (Shapiro–Wilk and Kolmogorov–Smirnov tests and Levene's test for homogeneity of variances) in order to guide bivariate test selection and to assess linear regression residuals. Bivariate comparisons between patients with EPDS scores <10 versus those with EPDS scores ≥10 were performed using *t*‐tests or non‐parametric equivalents (Mann–Whitney U test) for continuous variables. Chi‐square or Fisher's exact tests were performed for categorical variables, as appropriate.

To identify factors independently associated with the presence of PPD symptoms, we initially identified candidate variables based on a priori clinical relevance, existing evidence, and availability in our dataset. We then employed penalized logistic regression models [38] for final variable selection. Given the high dimensionality of predictors relative to sample size, we implemented three complementary modeling approaches: univariate logistic regression for each predictor to assess individual associations, L1‐penalized Least Absolute Shrinkage and Selection Operator (LASSO) logistic regression with all predictors for variable selection, and elastic net regression with combined L1 and L2 regularization (*α* = 0.5) to balance variable selection with coefficient shrinkage. The optimal regularization parameter (*C*) was selected through fivefold stratified cross‐validation using the area under the receiver operating characteristic curve (AUC) as the scoring metric. Variance inflation factors were calculated to assess multicollinearity among predictor variables for our models. For the penalized models, standard maximum likelihood–based confidence intervals are not directly available because regularization introduces intentional bias into coefficient estimates. Confidence intervals (CIs) were therefore estimated using nonparametric bootstrapping with 500 resamples, which is well‐established as sufficient for stable standard error estimation at our sample size, and statistical significance was assessed using the normal approximation method [39, 40]. Odds ratios (ORs) with 95% CI were calculated for all models. Variable selection stability was assessed by examining the consistency of variable inclusion across all three modeling approaches (univariate, LASSO, and elastic net). Variables retained consistently across multiple approaches were prioritized for inclusion in the final model, providing ensemble‐based stability assessment. Model performance was evaluated using AUC, sensitivity, specificity, and positive and negative predictive values.

For a priori planned sensitivity analyses, we repeated all analyses in the subset of patients with no prior history of anxiety or depression. Additionally, we performed linear regression analyses using continuous EPDS score as the outcome, employing both LASSO and elastic net regularization with the same cross‐validation approach. All statistical analyses were performed using Python (version 3.11.5). Results were independently validated using Stata MP v18.5 (StataCorp) to ensure reproducibility. For all machine learning models, we used an 80/20 stratified split evaluated across five different random seeds, with cross‐validation for hyperparameter tuning performed on the 80% training set. Final performance was assessed on the held‐out 20% test set. Statistical significance was defined as *p* < 0.05 for primary analyses. All tests were two‐sided.

## 3 RESULTS

Among 6712 eligible patients, 2551 (38%) were excluded because they did not have any postpartum EPDS scores recorded in our system. Our final cohort included 4161 pregnant individuals hospitalized between March 2012 and March 2025 for a variety of medical and obstetric complications. A total of 1864 patients (44.8%) delivered during their antepartum hospitalization. Upon admission, mean maternal age was 30.5 ± standard deviation (SD) 6.2 years and mean gestational age was 28.6 ± SD 6.9 weeks. Median hospitalization duration was 6 days (interquartile range [IQR], 3–9 days). The most common medical complications included chronic hypertension (632, 15%) and pregestational diabetes (425, 10%). Notably, the majority (2402, 58%) of participants had multiple chronic medical conditions, defined as two or more of the following conditions: asthma, hypertension, arthritis, diabetes, thyroid disorders, migraines, gastrointestinal disorders, cancer, seizure disorders, heart failure, other heart disease, or a physical disability [41]. The most common obstetric complications were preeclampsia spectrum disorders (778, 19%), preterm prelabor rupture of membranes (581, 14%), and complicated multiple gestation (442, 11%). It was not possible to accurately determine the primary reason for hospitalization from the discrete data fields used in this analysis. Overall, 1451 (35%) had a history of depression or anxiety preceding the antepartum hospitalization. A minority of patients (460, 11%) received pharmacologic treatment for anxiety or depression during their antepartum hospitalization. A total of 376 (9%) of our cohort had an inpatient psychiatric consultation ordered during their antepartum hospitalization. Data were complete for all variables except for preterm labor, which was missing for 55 individuals.

In our cohort, 1291 (31%) reported the presence of PPD symptoms (EPDS score ≥10). The distribution of EPDS scores was substantially right‐skewed and non‐normal (Figure S1). Additional cohort characteristics stratified by PPD symptoms are presented in Table 1. Multiple characteristics varied by EPDS score in bivariate analyses. Individuals with postpartum EPDS score ≥10 were admitted at a slightly earlier gestational age, as compared to those with EPDS score <10. Among individuals with EPDS score ≥10, there were higher proportions of Black individuals and those with government‐funded insurance, and lower proportions of multiparous patients, those identifying as Hispanic or Latino, and those reporting Spanish as their primary language, as compared to those with EPDS score <10. As expected, among those with EPDS score ≥10, there were substantially higher proportions of individuals with prior anxiety or depression diagnoses and receipt of pharmacologic treatment for anxiety or depression during antepartum hospitalization. Additionally, among those with EPDS score ≥10, a higher proportion of individuals had pregestational diabetes, chronic hypertension, and multiple chronic medical conditions. There were no significant differences in pregnancy complications by EPDS score.

**TABLE 1:** Characteristics of hospitalized antepartum patients stratified by postpartum depression symptoms (*n* = 4161).

Characteristics	Overall (*n* = 4161)	EPDS < 10 (*n* = 2870)	EPDS ≥ 10 (*n* = 1291)	*p* value
Maternal age (years)	30.5 ± 6.2	30.5 ± 6.2	30.3 ± 6.2	0.45
Gestational age on admission (weeks)	28.6 ± 6.9	28.8 ± 6.9	28.2 ± 7.0	**0.02**
Hospitalization length (days)	6.0 (3.0–9.0)	6.0 (3.0–9.0)	5.0 (3.0–9.0)	0.53
Multiparous	1670 (40.1)	1200 (41.8)	470 (36.4)	**0.001**
Race				**0.03**
White	2713 (65.2)	1898 (66.1)	815 (63.1)	
Black	1164 (28.0)	766 (26.7)	398 (30.8)	
Asian	219 (5.3)	157 (5.5)	62 (4.8)	
Other	65 (1.6)	49 (1.7)	16 (1.2)	
Hispanic or Latino ethnicity	1445 (34.7)	1046 (36.4)	399 (30.9)	**<0.001**
Spanish language	266 (6.4)	207 (7.2)	59 (4.6)	**0.002**
Marital status				**<0.001**
Married	2365 (56.8)	1692 (59.0)	673 (52.1)	
Single	1468 (35.3)	967 (33.7)	501 (38.8)	
Other	328 (7.9)	211 (7.4)	117 (9.1)	
Insurance type				**0.002**
Private	2300 (55.3)	1636 (57.0)	664 (51.4)	
Government	1797 (43.2)	1187 (41.4)	610 (47.3)	
Self‐pay	64 (1.5)	47 (1.6)	17 (1.3)	
Psychiatric history				
Prior anxiety or depression^a^	1451 (34.9)	753 (26.2)	698 (54.1)	**<0.001**
Pharmacologic treatment during hospitalization^b^	460 (11.1)	201 (7.0)	259 (20.1)	**<0.001**
Medical history				
Pregestational diabetes	425 (10.2)	263 (9.2)	162 (12.5)	**0.001**
Chronic hypertension	632 (15.2)	402 (14.0)	230 (17.8)	**0.002**
Multiple chronic medical conditions^c^	2402 (57.7)	1582 (55.1)	820 (63.5)	**<0.001**
Pregnancy complications				
Preterm prelabor rupture of membranes	581 (14.0)	389 (13.6)	192 (14.9)	0.28
Placenta accreta spectrum	153 (3.7)	100 (3.5)	53 (4.1)	0.37
Preeclampsia spectrum disorders	778 (18.7)	544 (19.0)	234 (18.1)	0.53
Gestational diabetes	333 (8.0)	226 (7.9)	107 (8.3)	0.69
Complicated multiple gestation	442 (10.6)	315 (11.0)	127 (9.8)	0.30

*Note*: Data presented as *n* (percentage), mean ± standard deviation, or median (interquartile range). *p* values calculated using Chi‐square, Fisher's exact, or Kruskal–Wallis tests, as appropriate.

Abbreviation: EPDS, Edinburgh Postnatal Depression Scale.

Bolded *p* values indicate statistical significance, defined as *p* < 0.05.

^a^ Prior anxiety or depression defined as any previous diagnoses or treatment preceding antepartum hospitalization.

^b^ Pharmacologic treatment defined as treatment with oral antianxiety or antidepression medications during antepartum hospitalization.

^c^ Multiple chronic medical conditions defined as two or more of the following diagnoses: asthma, hypertension, arthritis, diabetes, thyroid disorders, migraines, gastrointestinal disorders, cancer, seizure disorders, heart failure, other heart disease, or a physical disability.

**FIGURE 1:**
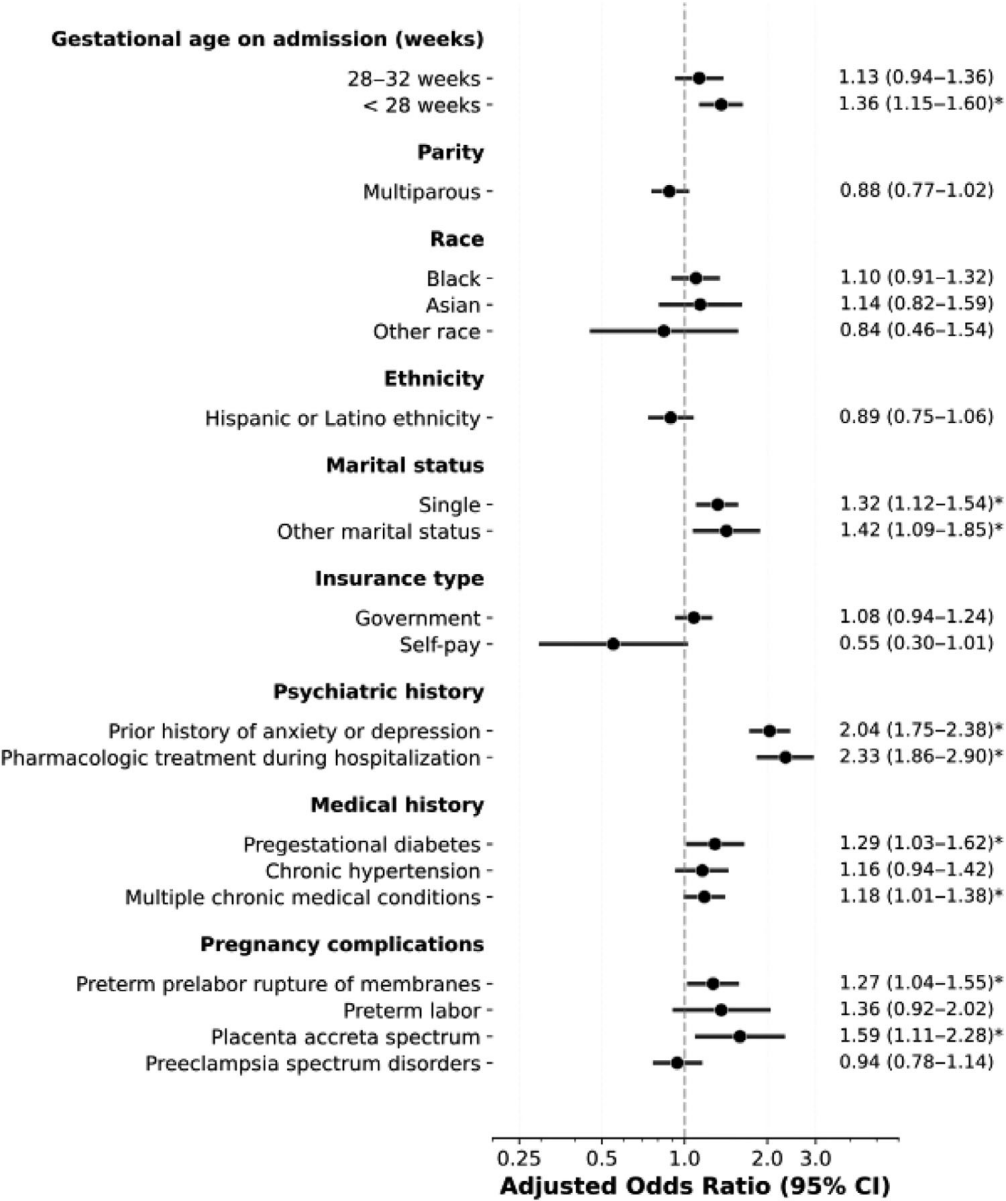
Forest plot of adjusted odds ratios from the final multivariable logistic regression model predicting postpartum depression symptoms following antepartum hospitalization (*n* = 4106). All variables listed are included simultaneously as covariates in a multivariable logistic regression model predicting postpartum depression symptoms, defined as Edinburgh Postnatal Depression Scale (EPDS) score ≥ 10. Bolded subheadings indicate variable groupings. Note the following reference categories for variables with >2 categories: gestational age: >32 weeks; race: White; marital status: married; insurance type: private. CI, confidence interval.

**FIGURE 2:**
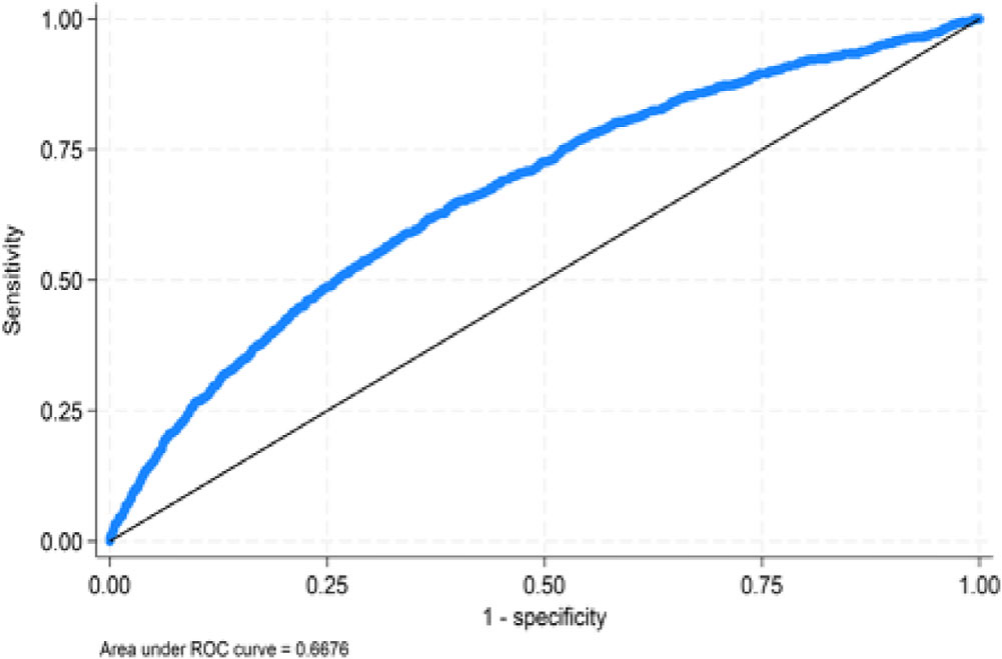
Receiver operating characteristic (ROC) curve for multivariable logistic regression model predicting postpartum depression symptoms following antepartum hospitalization (*n* = 4106). Area under the ROC curve (AUC) = 0.67 (95% CI, 0.65–0.69). Sensitivity 19%, specificity 94%, positive predictive value 58%, and negative predictive value 72%.

After variable selection following the methodology described above, and considering the candidate variables listed in Table 1, our final multivariable model included all variables listed in Table 2. For categorical variables, the reference group was assigned to the group predicted to be at the lowest risk for PPD symptoms. All variance inflation factors (VIFs) were below the threshold of 5.0, with mean VIF of 1.25, indicating acceptable levels of multicollinearity in our multivariable model (Table S1) [42]. Multivariable logistic regression modeling showed that the following clinical and demographic factors were significantly associated with presence of PPD symptoms (presented from highest to lowest strength of association): pharmacologic treatment for anxiety or depression during antepartum hospitalization (adjusted odds ratio [aOR], 2.33; 95% CI, 1.86–2.90), prior anxiety or depression diagnoses (aOR, 2.04; 95% CI, 1.75–2.38), placenta accreta spectrum (aOR, 1.59; 95% CI, 1.11–2.28), marital status of other (aOR, 1.42; 95% CI, 1.09–1.85) or single (aOR, 1.32; 95% CI, 1.12–1.54) as compared to married, admission gestational age <28 weeks (aOR, 1.36; 95% CI, 1.15–1.60, as compared to gestational age >32 weeks), pregestational diabetes (aOR, 1.29; 95% CI, 1.03–1.62), preterm prelabor rupture of membranes (aOR, 1.27; 95% CI, 1.04–1.55), and multiple chronic medical conditions (aOR, 1.18; 95% CI, 1.01–1.38). See Table 2 and Figure 1 for further details and results from the univariate and multivariable models. Discriminatory ability of the final multivariable model was fair; with area under the curve (AUC) of 0.67 (95% CI, 0.65–0.69), positive predictive value of 58%, and negative predictive value of 72% (Figure 2).

**TABLE 2:** Univariate and multivariable logistic regression models predicting postpartum depression symptoms following antepartum hospitalization (*n* = 4106).

	Unadjusted OR	Unadjusted 95% CI	Adjusted OR	Adjusted 95% CI
Gestational age on admission (weeks)				
>32 weeks	Reference	Reference	Reference	Reference
28–32 weeks	1.06	0.89–1.26	1.13	0.94–1.36
<28 weeks	**1.22**	1.05–1.42	**1.36**	1.15–1.60
Multiparous	**0.80**	0.70–0.91	0.88	0.77–1.02
Race				
White	Reference	Reference	Reference	Reference
Black	**1.21**	1.05–1.40	1.10	0.91–1.32
Asian	0.92	0.68–1.25	1.14	0.82–1.59
Other	0.76	0.43–1.35	0.84	0.46–1.54
Hispanic or Latino ethnicity	0.78	0.68–0.90	0.89	0.75–1.06
Marital status				
Married	Reference	Reference	Reference	Reference
Single	**1.30**	1.13–1.50	**1.32**	1.12–1.54
Other	**1.39**	1.09–1.78	**1.42**	1.09–1.85
Insurance type				
Private	Reference	Reference	Reference	Reference
Government	1.13	0.99–1.28	1.08	0.94–1.24
Self‐pay	**0.54**	0.30–0.98	0.55	0.30–1.01
Psychiatric history				
Prior history of anxiety or depression^a^	**2.49**	2.17–2.85	**2.04**	1.75–2.38
Pharmacologic treatment during hospitalization^b^	**3.33**	2.74–4.06	**2.33**	1.86–2.90
Medical history				
Pregestational diabetes	**1.42**	1.16–1.75	**1.29**	1.03–1.62
Chronic hypertension	**1.33**	1.11–1.59	1.16	0.94–1.42
Multiple chronic medical conditions^c^	**1.42**	1.24–1.62	**1.18**	1.01–1.38
Pregnancy complications				
Preterm prelabor rupture of membranes	1.11	0.92–1.34	**1.27**	1.04–1.55
Preterm labor	1.31	0.90–1.91	1.36	0.92–2.02
Placenta accreta spectrum	1.19	0.84–1.67	**1.59**	1.11–2.28
Preeclampsia spectrum disorders	0.95	0.80–1.12	0.94	0.78–1.14

*Note*: All models predict postpartum depression symptoms, defined as Edinburgh Postnatal Depression Scale (EPDS) score ≥10. Adjusted models include all listed variables as covariates.

Abbreviations: CI, confidence interval; OR, odds ratio.

Bolded values indicate statistical significance, defined as 95% CI not crossing one.

^a^ Prior history of anxiety or depression defined as any previous diagnoses or treatment preceding antepartum hospitalization.

^b^ Pharmacologic treatment defined as treatment with oral antianxiety or antidepression medications during antepartum hospitalization.

^c^ Multiple chronic medical conditions defined as two or more of the following diagnoses: asthma, hypertension, arthritis, diabetes, thyroid disorders, migraines, gastrointestinal disorders, cancer, seizure disorders, heart failure, other heart disease, or a physical disability.

In sensitivity analyses where models were stratified by prior mental health history, the direction of associations for key predictors remained consistent, though the discriminatory ability was reduced in the subset of patients (*n* = 2312) without prior mental health history (AUC 0.59 vs. 0.67 in full cohort), highlighting the importance of mental health history in risk prediction in our cohort (Table S2 and Figure S2). Linear regression analyses treating EPDS score as a continuous outcome confirmed the robustness of our main findings, with consistent identification of prior mental health history and early gestational age as significant predictors (Table S3). Finally, sensitivity analysis using alternative EPDS threshold of ≥13 yielded consistent results (see Table S4).

## 4 DISCUSSION

In our cohort from a single regional referral center, one in three individuals hospitalized during pregnancy subsequently reported presence of depression symptoms during the postpartum period. This study identified a set of specific demographic and clinical factors that were significantly associated with PPD symptoms, yet the overall ability to accurately identify those at increased risk was only fair. Pharmacologic treatment for anxiety or depression during antepartum hospitalization had the strongest association with presence of PPD symptoms within 8 weeks of delivery.

We found increased prevalence (one in three) for PPD symptoms in our hospitalized antepartum cohort, as compared to one in five prevalence in the general obstetric population [43, 44]. Prior studies have documented multiple social and biological risk factors for perinatal depression [45, 46], though relatively few studies have focused on hospitalized antepartum patients. A modern meta‐analysis [14] calculated that one in three hospitalized antepartum patients report symptoms of depression or anxiety during their hospital stay. While a small number of longitudinal studies have reported conflicting findings regarding the trajectory of depression symptoms during and after antepartum hospitalization [47, 48, 49], our study (*n* = 4161) is the largest to date demonstrating an increased risk of depression symptoms postpartum compared to the general obstetric population. The largest prior study (that we are aware of) assessed depression symptoms in 279 hospitalized antepartum patients in Israel, finding that approximately one in three hospitalized antepartum patients screened positive for depression (EPDS score ≥10) during their hospital stay, consistent with our postpartum findings [50].

Machine learning approaches have previously been utilized to predict adverse mental health outcomes, including progression of schizophrenia and bipolar disorder [51] and involuntary psychiatric admission [52]. Specific to perinatal mental health, a recent publication [53] presented results from a machine learning model leveraging variables routinely documented during prenatal care and delivery hospitalization to predict PPD among a large cohort (*n* = 29,168) without history of depressive symptoms. Their model demonstrated improved discriminatory ability as compared to our multivariable regression model, with an AUC of 0.75 (compared to our AUC of 0.67). Prior studies predicting PPD risk have achieved further improved discriminatory ability (AUCs of 0.80–0.90) but have limitations related to generalizability [54, 55] and the finding that history of depression drives much of the discriminatory performance [54, 56]. To contextualize the interpretation of AUC values, they can range from 0.5 to 1.0, with 0.5 indicating chance discrimination and 1.0 indicating perfect discrimination. While AUC values above 0.7–0.8 are often considered clinically useful, labeling of AUC values (i.e., poor, moderate, good, and excellent) is arbitrary, and high discriminatory ability is not sufficient to ensure positive effects from deploying prediction models in clinical practice [57]. Consistent with our findings, all prior studies predicting PPD have found that varying aspects of mental health history (i.e., anxiety disorders and prenatal EPDS score [53]; depression and anxiety history, recent psychiatric medication use [54]; psychiatric history [55]; anxiety, depression, and antidepressants during pregnancy [56]) are the strongest predictors of PPD.

We found that one in three individuals who had an antepartum hospitalization went on to report PPD symptoms, and yet our ability to accurately identify those at increased risk based on clinical and demographic factors known during antepartum hospitalization was only fair using multivariable modeling. In our full model, the strongest predictive factors included pharmacologic treatment for anxiety and depression during antepartum hospitalization and history of anxiety or depression preceding antepartum hospitalization. One might predict that those receiving mental health treatment during antepartum hospitalization would have improved outcomes, given that they are engaged in treatment. The fact that these patients are in fact at *increased* risk for PPD symptoms highlights the profound inadequacies of our current perinatal mental health treatment system, where the small minority of patients receive adequate treatment and achieve remission [58]. Additionally, other demographic and clinical factors remained independently associated with the presence of PPD symptoms, suggesting that clinical context and non‐medical drivers of health remain important contributors to postpartum mental health outcomes.

Additionally, almost half (46%) of individuals with PPD symptoms in our study had no prior history of anxiety or depression, highlighting the potential impact of high‐risk pregnancy and antepartum hospitalization on perinatal mental health. Collectively, these findings support universal screening for PPD and provision of tailored mental health services within the high‐risk population of those experiencing antepartum hospitalization. Future research should focus on dissemination and implementation of proven methods, including comprehensive, system‐wide models that integrate screening and tailored mental health support (such as the collaborative care model [59]). In addition, the US Preventive Services Task Force (USPSTF) recommends evidence‐based behavioral interventions to prevent perinatal depression, including Mothers and Babies (MB) and Reach Out, Stay Strong Essentials (ROSE) [60]. Despite USPSTF recommendations to provide these evidence‐based interventions to individuals at risk for perinatal depression [60], there are no studies of MB, ROSE, or similar preventive interventions in hospitalized antepartum patients. This patient population faces specific stressors that require tailored coping strategies, such as prolonged separation from family during antepartum hospitalization or an infant with medical complications receiving care in the neonatal intensive care unit. With careful adaptation, piloting, and evaluation, these established preventive strategies could be beneficial for patients during and after antepartum hospitalization. Further studies are needed to determine the role of such preventive interventions in supporting mental health in high‐risk patients experiencing antepartum hospitalization.

It is important to note that any predictive modeling developed for use in healthcare settings can reflect biases that may perpetuate systemic inequities [61]. Physician bias in provision of care, as well as systemic barriers that prevent certain groups of people from accessing care, can lead to biased predictive models. This is especially relevant for perinatal mental health, as there are many psychosocial and environmental factors [31] contributing to mental health which may not be captured in the demographic and clinical data used to develop the models. As these types of predictive models are further refined for potential use in clinical contexts, future research should focus on evaluating and mitigating bias [62].

Our study has notable strengths. It is a large cohort (*n* = 4161) for this specific perinatal population (hospitalized antepartum patients) that links hospitalization data with PPD screening performed in the outpatient setting. Our study also included a representative patient population reflecting the diversity of our surrounding community and our center's role as a regional referral center. Notably, our sample includes a large number of individuals of Hispanic ethnicity, including Spanish language patients, enhancing the representativeness of our findings. We utilized comprehensive data from our electronic health record, including demographics, medical and obstetric history, hospitalization characteristics, and postpartum EPDS scores. Our statistical approach was robust, using modern biostatistical methodology and independent validation of results.

Our study also has important limitations that should be noted. Our study cohort includes patients from a single center, thus limiting generalizability to other populations with differing characteristics. We did not have data on smoking and substance use for our cohort, which may have introduced residual confounding given the association between substance use disorders and mental health conditions. We recognize limitations in defining our primary outcome as EPDS score ≥10; this lower threshold (as compared to the other commonly used EPDS threshold of ≥13) increases sensitivity while decreasing specificity for the detection of depression. Notably, our sensitivity analysis using the alternative EPDS threshold of ≥ 13 demonstrated consistent findings. Additionally, much of the clinical data extracted from our electronic health record is based on diagnosis codes, and the possibility of misclassification exists. The effects of missed or inaccurate diagnoses are challenging to predict, and this may have introduced bias into our prediction models. Furthermore, given the high comorbidity and overlapping symptom profiles of anxiety and depression during the perinatal period, we elected to combine these diagnoses into a composite variable in our analyses, limiting our ability to draw conclusions about individual risks associated with these distinct diagnoses. Future studies with comprehensive linked delivery and neonatal outcome data, in addition to a robust composite PPD variable (i.e., EPDS score, diagnosis codes, postpartum medications, and emergency department utilization), would enable a fuller understanding of the causal pathways between antepartum hospitalization and postpartum mental health.

Another notable limitation is related to the creation of our study cohort. A significant proportion (38%) of those eligible for study inclusion did not have any postpartum EPDS scores in our system; their postpartum mental health outcomes remain unknown. It is well‐established that a significant proportion of postpartum women do not attend any postpartum visits [63]. Attendance is lower among marginalized populations who may have limited resources [64, 65]; these populations are also at increased risk for perinatal mental health conditions [9, 66]. This creates a systematic selection bias, where patients at increased risk for PPD were less likely to be included in our study cohort. Thus, our reported 31% prevalence of PPD symptoms is likely underestimated, in addition to the underestimation of the strength of associations between demographic risk factors and PPD symptoms. Future studies should employ a prospective design with active follow‐up protocols or linked administrative claims data to capture PPD outcomes in patients who do not attend outpatient postpartum visits. Finally, our study has limitations inherent to all retrospective studies, including the possibility of unmeasured bias and confounding. Our findings are primarily hypothesis‐generating and cannot determine causality.

## 5 CONCLUSIONS

One in three individuals who experienced antepartum hospitalization went on to report PPD symptoms. This highlights the high‐risk nature of this cohort and the importance of comprehensive postpartum follow‐up. We identified that certain demographic and clinical factors were associated with the presence of PPD symptoms in our study, yet the overall ability to accurately identify those at increased risk was only fair. These findings support the need for universal mental health service provision for the high‐risk population of individuals who experience antepartum hospitalization.

## AUTHOR CONTRIBUTIONS

**Alison N. Goulding**: Conceptualization (lead); funding acquisition (lead); methodology (supporting); resources (co‐lead); writing—original draft (lead); writing—review and editing (lead). **Daniel Palacios**: Data curation (supporting); formal analysis (lead); methodology (co‐lead); project administration (co‐lead); visualization (lead); writing—original draft (supporting); writing—reviewing and editing (supporting). **Sukru Aras**: Formal analysis (supporting); methodology (co‐lead); visualization (supporting); writing—reviewing and editing (supporting). **Hu Chen**: Project administration (co‐lead); methodology (supporting); supervision (supporting); writing—reviewing and editing (supporting). **Sasidhar Pasupuleti**: Data curation (lead); writing—review and editing (supporting). **Marika Toscano**: Conceptualization (supporting); writing—review and editing (supporting). **Nicole Cirino**: Conceptualization (supporting); writing—review and editing (supporting). **Israel C. Christie**: Formal analysis (supporting); methodology (supporting); writing—reviewing and editing (supporting). **Zhandong Liu**: Methodology (supporting); resources (co‐lead); writing—review & editing (supporting). **Emily S. Miller**: Conceptualization (supporting); methodology (supporting); writing—review and editing (supporting). **Terri L. Fletcher**: Conceptualization (supporting); methodology (supporting); supervision (supporting); writing—original draft (supporting); writing—review and editing (supporting).

## CONFLICT OF INTEREST STATEMENT

The authors declare no conflicts of interest.

## ETHICS STATEMENT

This study was approved by the Baylor College of Medicine Institutional Review Board (H‐54811).

## PATIENT CONSENT STATEMENT

A waiver of consent was approved by the Baylor College of Medicine Institutional Review Board (H‐54811).

## Supporting information



Supporting Information

## Data Availability

The data that support the findings of this study are available from the corresponding author upon reasonable request.
