# Supplementary material for: Factors associated with postpartum depression symptoms following antepartum hospitalization
Source: Pregnancy (Hoboken). 2026 May 22;2(3):e70319. doi: 10.1002/pmf2.70319 (PMC13344247; doi:10.1002/pmf2.70319)
Supplement: Supplementary file 1 [file PMF2-2-e70319-s001.docx]

**SUPPLEMENTARY TABLES AND FIGURES**

**Table S1.** Variance Inflation Factors (VIF) for the multivariable logistic regression model predicting postpartum depression symptoms among hospitalized antepartum patients (n = 4,106).

| **Variable** | **VIF** | **1/VIF (Tolerance)** |
| --- | --- | --- |
| Gestational age on admission |  |  |
| 28–32 weeks | 1.69 | 0.591 |
| < 28 weeks | 1.80 | 0.557 |
| Marital status |  |  |
| Other | 1.09 | 0.920 |
| Single | 1.21 | 0.827 |
| Race |  |  |
| Black | 1.54 | 0.651 |
| Asian | 1.13 | 0.884 |
| Other | 1.03 | 0.970 |
| Hispanic or Latino ethnicity | 1.41 | 0.710 |
| Insurance type |  |  |
| Government | 1.03 | 0.967 |
| Self-pay | 1.04 | 0.966 |
| **Psychiatric history** | | |
| Prior anxiety or depression | 1.25 | 0.800 |
| Pharmacologic treatment | 1.22 | 0.818 |
| **Medical history** | | |
| Pregestational diabetes | 1.07 | 0.938 |
| Chronic hypertension | 1.27 | 0.786 |
| Multiple chronic conditions | 1.28 | 0.779 |
| **Pregnancy complications** | | |
| Preterm labor | 1.04 | 0.958 |
| Preterm prelabor rupture of membranes | 1.25 | 0.797 |
| Placenta accreta spectrum | 1.09 | 0.920 |
| Preeclampsia spectrum disorders | 1.35 | 0.740 |
| **Mean VIF** | 1.25 |  |

VIF, Variance Inflation Factor.

All VIF values are below the threshold of 5.0, indicating no substantial multicollinearity among the predictor variables in the model. Reference categories for categorical variables are not shown (gestational age > 32 weeks, White race, married, private insurance).

**Table S2.** Multivariable logistic regression model predicting postpartum depression symptoms among hospitalized antepartum patients without prior mental health history (n = 2,312).

| **Predictor Variables** | **Odds Ratio** | **OR (95% CI)** | ***P* value** |
| --- | --- | --- | --- |
| Gestational age on admission |  |  |  |
| > 32 weeks | Reference | Reference |  |
| 28–32 weeks | 1.16 | 1.16 (0.87–1.55) | 0.308 |
| < 28 weeks | **1.72** | 1.73 (1.35–2.20) | < 0.001 |
| Multiparous | 0.99 | 0.99 (0.81–1.22) | 0.947 |
| Race |  |  |  |
| White | Reference | Reference |  |
| Black | 1.19 | 1.19 (0.90–1.58) | 0.233 |
| Asian | 1.50 | 1.50 (0.99–2.26) | 0.055 |
| Other | 0.51 | 0.51 (0.21–1.22) | 0.131 |
| Hispanic or Latino ethnicity | 0.86 | 0.86 (0.65–1.12) | 0.256 |
| Marital status |  |  |  |
| Married | Reference | Reference |  |
| Other | 1.11 | 1.11 (0.75–1.66) | 0.600 |
| Single | **1.26** | 1.26 (1.00–1.59) | 0.047 |
| Insurance type |  |  |  |
| Private | Reference | Reference |  |
| Government | 1.16 | 1.16 (0.95–1.41) | 0.158 |
| Self-pay | **0.35** | 0.35 (0.12–0.99) | 0.048 |
| **Medical history** | | | |
| Pregestational diabetes | **1.44** | 1.44 (1.05–1.97) | 0.023 |
| Chronic hypertension | 0.98 | 0.98 (0.72–1.33) | 0.913 |
| Multiple chronic conditions^1^ | **1.29** | 1.29 (1.03–1.62) | 0.027 |
| **Pregnancy complications** | | | |
| Preterm labor | 1.01 | 1.01 (0.57–1.81) | 0.965 |
| Preterm prelabor rupture of membranes | 1.18 | 1.18 (0.90–1.55) | 0.241 |
| Placenta accreta spectrum | 0.94 | 0.94 (0.55–1.61) | 0.821 |
| Preeclampsia spectrum disorders | 0.82 | 0.82 (0.61–1.08) | 0.157 |

OR, odds ratio; CI, confidence interval. Model predicts postpartum depression symptoms, defined as Edinburgh Postnatal Depression Scale score ≥ 10. All listed variables are included as covariates.

^1^Multiple chronic medical conditions defined as two or more of the following diagnoses: asthma, hypertension, arthritis, diabetes, thyroid disorders, migraines, gastrointestinal disorders, cancer, seizure disorders, heart failure, other heart disease, or a physical disability.

**Table S3.** Multivariable linear regression model predicting postpartum depression symptoms among hospitalized antepartum patients (n = 4,106).

| **Predictor Variables** | **Coefficient** | **Coefficient (95% CI)** | ***P* value** |
| --- | --- | --- | --- |
| Gestational age on admission |  |  |  |
| > 32 weeks | Reference | Reference |  |
| 28–32 weeks | 0.25 | 0.25 (−0.22 to 0.72) | 0.304 |
| < 28 weeks | **0.77** | 0.77 (0.35 to 1.19) | < 0.001 |
| Multiparous | −0.02 | −0.02 (−0.38 to 0.35) | 0.932 |
| Race |  |  |  |
| White | Reference | Reference |  |
| Black | 0.15 | 0.15 (−0.34 to 0.65) | 0.544 |
| Asian | 0.66 | 0.66 (−0.17 to 1.50) | 0.121 |
| Other | −0.12 | −0.12 (−1.58 to 1.34) | 0.874 |
| Hispanic or Latino ethnicity | **−0.59** | −0.59 (−1.04 to −0.14) | 0.010 |
| Marital status |  |  |  |
| Married | Reference | Reference |  |
| Other | **1.06** | 1.06 (0.37 to 1.76) | 0.003 |
| Single | **1.04** | 1.04 (0.62 to 1.45) | < 0.001 |
| Insurance type |  |  |  |
| Private | Reference | Reference |  |
| Government | 0.03 | 0.03 (−0.33 to 0.39) | 0.861 |
| Self-pay | −1.09 | −1.09 (−2.44 to 0.25) | 0.112 |
| **Psychiatric history** | | | |
| Prior anxiety or depression¹ | **1.58** | 1.58 (1.18 to 1.98) | < 0.001 |
| Pharmacologic treatment² | **3.10** | 3.10 (2.70 to 3.50) | < 0.001 |
| **Medical history** | | | |
| Pregestational diabetes | 0.51 | 0.51 (−0.10 to 1.12) | 0.101 |
| Chronic hypertension | 0.22 | 0.22 (−0.32 to 0.77) | 0.419 |
| Multiple chronic conditions³ | **0.52** | 0.52 (0.12 to 0.92) | 0.011 |
| **Pregnancy complications** | | | |
| Preterm labor | **1.47** | 1.47 (0.42 to 2.52) | 0.006 |
| Preterm prelabor rupture of membranes | 0.28 | 0.28 (−0.25 to 0.81) | 0.295 |
| Placenta accreta spectrum | 0.64 | 0.64 (−0.31 to 1.60) | 0.186 |
| Preeclampsia spectrum disorders | −0.07 | −0.07 (−0.55 to 0.42) | 0.787 |

CI, confidence interval. Model predicts postpartum depression symptoms (Edinburgh Postnatal Depression Scale score, continuous variable). All listed variables are included as covariates.

¹Prior anxiety or depression defined as any previous diagnoses or treatment preceding antepartum hospitalization.

²Pharmacologic treatment defined as treatment with oral antianxiety or antidepression medications during antepartum hospitalization.

³Multiple chronic medical conditions defined as two or more of the following diagnoses: asthma, hypertension, arthritis, diabetes, thyroid disorders, migraines, gastrointestinal disorders, cancer, seizure disorders, heart failure, other heart disease, or a physical disability.

**Table S4.** Multivariable linear regression model predicting postpartum depression symptoms among hospitalized antepartum patients at alternative EPDS thresholds (n = 4,106).

|  | **EPDS ≥ 10** | | **EPDS ≥ 13** | |
| --- | --- | --- | --- | --- |
|  | Adjusted OR | Adjusted 95% CI | Adjusted OR | Adjusted 95% CI |
| Gestational age on admission (weeks) |  |  |  |  |
| > 32 weeks | Reference | Reference | Reference | Reference |
| 28-32 weeks | 1.13 | 0.94–1.36 | 1.01 | 0.81–1.25 |
| < 28 weeks | **1.36** | 1.15–1.60 | **1.24** | 1.02–1.50 |
| Multiparous | 0.88 | 0.77–1.02 | 0.95 | 0.80–1.13 |
| Race |  |  |  |  |
| White | Reference | Reference | Reference | Reference |
| Black | 1.10 | 0.91–1.32 | 1.11 | 0.89–1.38 |
| Asian | 1.14 | 0.82–1.59 | **1.47** | 1.01–2.15 |
| Other | 0.84 | 0.46–1.54 | 0.68 | 0.30–1.54 |
| Hispanic or Latino ethnicity | 0.89 | 0.75–1.06 | 0.95 | 0.78–1.17 |
| Marital status |  |  |  |  |
| Married | Reference | Reference | Reference | Reference |
| Single | **1.32** | 1.12–1.54 | **1.53** | 1.27–1.84 |
| Other | **1.42** | 1.09–1.85 | **1.53** | 1.14–2.07 |
| Insurance type |  |  |  |  |
| Private | Reference | Reference | Reference | Reference |
| Government | 1.08 | 0.94–1.24 | 1.07 | 0.91–1.25 |
| Self-pay | 0.55 | 0.30–1.01 | 0.72 | 0.37–1.42 |
| **Psychiatric history** |  |  |  |  |
| Prior history of anxiety or depression^1^ | **2.04** | 1.75–2.38 | **3.06** | 2.56–3.65 |
| Pharmacologic treatment during hospitalization^2^ | **2.33** | 1.86-2.90 | **1.68** | 1.41–2.01 |
| **Medical history** |  |  |  |  |
| Pregestational diabetes | **1.29** | 1.03–1.62 | 1.26 | 0.97–1.63 |
| Chronic hypertension | 1.16 | 0.94–1.42 | 1.08 | 0.86–1.37 |
| Multiple chronic medical conditions^3^ | **1.18** | 1.01–1.38 | 1.13 | 0.94–1.36 |
| **Pregnancy complications** |  |  |  |  |
| Preterm prelabor rupture of membranes | **1.27** | 1.04–1.55 | 1.01 | 0.79–1.29 |
| Preterm labor | 1.36 | 0.92–2.02 | **1.64** | 1.06–2.52 |
| Placenta accreta spectrum | **1.59** | 1.11–2.28 | 1.11 | 0.72–1.73 |
| Preeclampsia spectrum disorders | 0.94 | 0.78–1.14 | 0.93 | 0.75–1.17 |

EPDS, Edinburgh Postnatal Depression Scale; OR, odds ratio; CI, confidence interval.

Models predict postpartum depression symptoms, defined as EPDS score ≥ 10 or EPDS score ≥ 13. All listed variables are included as covariates.

^1^Prior history of anxiety or depression defined as any previous diagnoses or treatment preceding antepartum hospitalization.

^2^Pharmacologic treatment defined as treatment with oral antianxiety or antidepression medications during antepartum hospitalization.

^3^Multiple chronic medical conditions defined as two or more of the following diagnoses: asthma, hypertension, arthritis, diabetes, thyroid disorders, migraines, gastrointestinal disorders, cancer, seizure disorders, heart failure, other heart disease, or a physical disability.

**Figure S1.** Distribution of postpartum Edinburgh Postnatal Depression Scale (EPDS) scores in a cohort who previously experienced antepartum hospitalization (n = 4,161).

Histogram of EPDS scores with normal curve overlaid for reference. All EPDS scores were collected in the postpartum period, within 8 weeks of delivery. The distribution is substantially right-skewed, with a peak at lower scores.

**Figure S2.** Receiver operating characteristic (ROC) curve for multivariable logistic regression models predicting postpartum depression symptoms (EPDS ≥ 10) following antepartum hospitalization for patients without prior mental health history (n = 2,312).

Area under the receiver operating characteristic curve (AUC) = 0.59, as compared to AUC = 0.67 in the full cohort. The reduced discriminative ability in this subgroup highlights the importance of mental health history in risk prediction.
